# Simvastatin Attenuates Oxidative Stress, NF-κB Activation, and Artery Calcification in LDLR-/- Mice Fed with High Fat Diet via Down-regulation of Tumor Necrosis Factor-α and TNF Receptor 1

**DOI:** 10.1371/journal.pone.0143686

**Published:** 2015-12-01

**Authors:** Chih-Pei Lin, Po-Hsun Huang, Chung Fang Lai, Jaw-Wen Chen, Shing-Jong Lin, Jia-Shiong Chen

**Affiliations:** 1 Division of Central Laboratory, Department of Pathology and Laboratory Medicine, Taipei Veterans General Hospital, Taipei, Taiwan; 2 Department of Biotechnology and Laboratory Science in Medicine and Institute of Biotechnology in Medicine, Taipei, Taiwan; 3 Division of Cardiology, Department of Medical Research, Taipei Veterans General Hospital, Taipei, Taiwan; 4 Institute of Clinical Medicine, National Yang-Ming University, Taipei, Taiwan; 5 Volunteer Scientist, E-da Hospital, Kaohsiung, Taiwan; 6 Department of Internal Medicine, Taipei Veterans General Hospital, Taipei, Taiwan; 7 Institute of Pharmacology, National Yang-Ming University, Taipei, Taiwan; Brigham and Women's Hospital, Harvard Medical School, UNITED STATES

## Abstract

Simvastatin (SIM) is anti-inflammatory. We used low density lipoprotein receptor knockout (LDLR-/-) mice and human aortic smooth muscle cells (HASMCs) as model systems to study the effect of SIM on arterial calcification and to explore the potential mechanisms contributing to this protective effect. High-fat diet (HFD) caused the LRLR -/- to develop dyslipidemia, diabetics, atherosclerosis and aortic smooth muscle calcification. SIM, N-acetyl cysteine (NAC, a ROS scavenger) and apocynin (APO, a NADPH oxidase inhibitor) did not significantly retard the development of dyslipidemia or diabetic. However, those treatments were still effective in attenuating the HFD-induced atherosclerosis and aortic smooth muscle calcification. These findings suggest that the protective effect of SIM against aortic calcification is not contributed by the cholesterol lowering effect. SIM, NAC and APO were found to attenuate the HFD induced elevation of serum TNF-α, soluble TNFR1 (sTNFR1), 3-nitro-tyrosine. We hypothesized that the pro-inflammatory cytokine, oxidative stress and TNFR1 played a role in inducing aortic calcification. We used HASMC to investigate the role of TNF-α, oxidative stress and TNFR1 in inducing aortic calcification and to elucidate the mechanism contributes the protective effect of SIM against aortic calcification. We demonstrated that treating HASMC with TNF-α induced cell Ca deposit and result in an increase in ALP, NADPH oxidase activity, NF-kB subunit p65, BMP2, MSX2, and RUNX2 expression. SIM suppressed the TNF-α induced activation of NADPH oxidase subunit p47, the above-mentioned bone markers and TNFR1 expression. Furthermore, p65, p47 and TNFR1 siRNAs inhibited the TNF-α-mediated stimulation of BMP-2, MSX2, RUNX2 expression. SIM, APO, and NAC either partially inhibit or completely block the TNF-α induced H_2_O_2_ or superoxide production. These results suggest that SIM may, independent of its cholesterol-lowering effect, suppresses the progression of vascular diseases through the inhibition of the inflammation mediators TNF-α and TNFR1.

## Introduction

Artery calcification commonly occurs in patients with type II diabetes mellitus (T2DM), chronic renal disease or elderlies [1–3]. The resultant arterial stiffness leads to ischemia as well as various complications, such as early mortality, sudden death, vision loss, myocardial infarction, stroke and amputation [4]. Currently, no effective drugs are available for the prevention or treatment of vascular calcification. Many factors that have been linked to an increased prevalence of artery calcification are associated with elevated oxidative stress, including hypercholesterolemia, hypertension, and diabetes mellitus [5–7]. It is reported that the production of reactive oxygen species (ROS) by vascular cells lead to the development of calcific aortic valvular stenosis in humans [8]. Upon stimulation, oxidases in various compartments, including the endothelium, media, and adventitia, produce ROS which render vascular smooth muscle cells (VSMCs) under oxidative stress [9]. The oxidative stress may injure VSMC or perturbs the regulatory signal to induce the VSMC to differentiate into osteoblast which leads to artery calcification [10]. Recent studies have shown that VSMC expresses osteogenic markers, such as bone morphogenetic protein-2 (BMP2) and runt-related transcription factor-2 (RUNX2), and those osteogenic markers play important roles in vascular calcification [11, 12]. Msh homeobox 2 (MSX2) belongs to the homeobox transcription factor family and is involved in cranial bone development [13]. MSX2 promotes aortic valve and medial calcification *in vivo* by creating an osteogenic environment through the induction of the Wnt signaling pathway [14]. MSX2-deficient mice show defects in cranial bone formation, whereas transgenic mice over-expressing MSX2 exhibit an overall increase in bone volume [13, 15]. MSX2-Wnt signaling participates in aortic valve and medial calcification in LDLR-/- mice fed a high-fat diet (HFD) [16]. In addition, necrosis factor-alpha (TNF-α) has been found to play a crucial role in the calcification of vascular smooth muscle cells *in vitro* and *in vivo* [16, 17]. A subsequent study showed that tumor necrosis factor receptor 1 (TNFR1) activation leads to the production of ROS which upregulated the TNF-α expression and the elevation of TNF-α then activate MSX2 program to cause a pro-calcific response in mice aortic myofibroblast [18].

Alkaline phosphatase (ALP), which is a functional phenotypic marker of osteoblast and is often used as a molecular marker of vascular calcification [19]. Based on previous findings, inorganic phosphate (Pi) has been shown to be an important inducer of VSMC calcification, which is morphologically similar to that observed in calcified human heart valves and the aortic media. The transport of Pi into VSMCs has been suggested to play an important role in the initiation of extracellular matrix calcification [20]. Recently, structures that are similar to matrix vesicles derived from apoptotic VSMCs have been identified in human calcified arteries [21]. Atorvastatin has been demonstrated to reduce calcification using a human aortic smooth muscle cell model [22]. Data from two retrospective studies [23, 24] and one prospective study [25] were reported beneficial effect of statin treatments on aortic stenosis. However, the beneficial effect of statin cannot be confirmed in two prospective human clinical studies while both studies showed a significant reduction in cholesterol level [26, 27]. These conflicting findings may be due to different disease state of the patients, study design (type of statin used, length of treatment and dose), or method used to measure progression of stenosis (echocardiography or electron beam tomography). Despite the inconsistent reports on the effect of statin on human artery stenosis, the majority of the reports showed a positive effect on the progression of aortic stiffness [28, 29]. Rizosa et al reviewed literature reporting effect of statin on aortic and peripheral artery stiffness by pulse wave velocity. The authors found 6 out of the 9 human clinical studies showed statin is effective in reducing artery stiffness [28]. Based on these findings, we further speculate that SIM may attenuate the progression of artery calcification and stiffness. In this study, we used human artery smooth cell (HASMC) and high fat diet fed LDLR-/- mice to clarify the effect and mechanism of SIM on artery calcification.

## Materials and Methods

### Reagents

Human smooth muscle cell growth medium (M231), smooth muscle growth supplement, trypsin/EDTA solution, trypsin neutralizer solution and HASMCs were obtained from Cascade Biologics (Portland, OR). Fetal bovine serum (FBS), antibiotic-antimycotic mixture, mice TNF-α kits, oligofectamine and 2’,7’-dichlorofluorescein diacetate (DCFDA) were obtained from Life technology (Grand Island, NY). Small interfering RNA (siRNA) oligonucleotides against TNFR 1(sc-29507), p65 (sc-29410), and p47 (sc-76032) and antibodies against human TNFR1 (sc-8436), MSX-2(sc-17729), BMP2(sc-6895), RUNX2(sc-10758), CD68 (sc-9139), β-actin(sc-47778), and anti-hnRNA c1/c2 (sc-32308)were obtained from SantaCruz Biotechnology (Santa Cruz, CA). Antibodies against human NF-kB subunit P65(#558421), NADPH oxidase subunit p47(#610354), and caveoli-1 (610406) were obtained from BDBiosciences (San Jose, CA). Unless otherwise specified, all other chemicals and reagents were obtained from Sigma-Aldrich (St. Louis, MO).

### Animals

Male LDLR-/- mice (Jackson Labs #002207; C57BL/6J background) were fed a chow diet (Picolab Rodent Diet 20 #5053, PMI Nutrition International, St. Louis, MO) until 2 months of age, after which they were fed an HFD (Harlan Teklad Diet TD88137; 42% fat calories and 0.15% cholesterol) for 6 months. All mice were kept in microisolator cages under a 12-h day/night cycle. All experimental procedures and protocols involving animals were approved by the institutional animal care committee of National Yang-Ming University (Taipei, Taiwan) and complied with the Guide for the Care and Use of Laboratory Animals 117. The experimental design was randomized. All studies had 5 to 10 animals per treatment arm as indicated. To examine the effect of anti-aortic calcification in response to SIM (10 mg^-1^kg^-1^day^-1^) (BioVision Research Products, Milpitas, CA), N-acetyl-cysteine (250 mg^-1^kg^-1^day^-1^), or apocynin (40 mg^-1^kg^-1^day^-1^), these reagents were administered by intraperitoneal (IP) injection simultaneously with the HFD for 6 months. Metabolic parameters and the nitrotyrosine level were analyzed in serum samples isolated from each treatment group.

At 24 weeks, LDLR−/− mice were sacrificed by exsanguination under anesthesia (ketamine-HCl 100 mg/kg and xylazine 20 mg/kg via i.p. injection after over nigh fasting. Animals were considered as adequately anaesthetized when no attempt to withdraw the limb after pressure could be observed). The thoracic cavity was opened, a blood sample was collected, and then aorta was isolated. LDLR−/− mice aorta from heart to diaphragm was collected for artery calcification analysis by IHC method. At 20 weeks, wild type C57B6J mice were fasted overnight and anesthesia by ketamine/xylazine. Subsequently a blood sample was collected from the facial vein of mice.

### Histology and immunohistochemistry

Aorta from heart to diaphragm was collected, cut into 4 sections and processed for histological staining as described by Al-Aly Z et al [16]. For the histological staining of other tissues, paraffin sections (5 μm) through the dissenting aorta were obtained. Atherosclerotic lesions in the aortic artery were visualized by H&E staining, and calcium deposition in the aortic artery was identified by von Kossa and alizarin red S staining. Immunohistochemical staining of macrophages (CD68) and vascular smooth muscle cell actin (SM α-actin) was performed as previously described [30].

### Enzyme-linked immunosorbent assay (ELISA)

The levels of TNF-α, sTNFR1, and nitrotyrosine in circulation were determined by ELISA with mice TNF-α kits, mice sTNFR1 kits (R&D Systems, Minneapolis, MN) and an oxiselect nitrotyrosine ELISA kit (Cell Biolabs, San Diego, CA). The procedures were carried out according to the manufacturer’s instructions.

### Cell cultures and cell viability assay

HASMCs were from Life technology (Grand Island, NY. catalog number C-007-5C). Cells grown and passaged as described previously [31]. They were grown in M231 medium containing smooth muscle cell growth supplement and a 1% antibiotic-antimycotic mixture in an atmosphere of 95% air and 5% CO_2_ at 37°C in plastic flasks. At confluence, the cells were sub-cultured at a ratio of 1:3, and passage numbers 3 through 8 were used. After incubation with TNF-α, SIM, and calcium salt, cell viability was measured with the 3-(4,5-dimethylthiazol-2-yl)-2,5-diphenyl tetrazolium bromide (MTT) assay, showing consistent levels of greater than 95%. The calcification of HASMCs was induced *in vitro* by the addition of TNF-α into osteogenic media containing 10 mM β-glycerophosphate and 50 μg/mL ascorbic acid.

### Quantification of calcium deposition

Aorta from the arch to the diaphragm from each animal were dissected and analyzed for its calcium content analysis using a BioChain Calcium Kit (BioChain, Hayward, CA, USA) as previously described [32]. Briefly, cultured cells were washed twice with 250 μl PBS and demineralized with 250 μl of 0.6 N HCl for 12 h. A working reagent was prepared by mixing 75μl reagents A and 75μl regent B and was equilibrated to room temperature before use. A volume of 5 μl of diluted standards or samples was transferred into each well of a clear-bottom 96-well plate. Then, 200 μl of the working reagent was added, and the solution was mixed by light tapping. After incubation for 3 min at room temperature, absorbance was measured at 570–650 nm with a 96-well reader. The remaining cells were washed three times with 250 μl PBS and solubilized in 200 μl of lysis solution containing 0.1 N NaOH and 0.1% sodium dodecyl sulfate (SDS) at room temperature for 5 min. The protein concentration was measured with a Bio-Rad DC Protein Assay Kit. The calcium concentration was normalized to the total protein concentration of the whole cells. The aorta isolated form experimental mice and aortic segments were weighed and decalcified with 0.6 N HCl for 24 hrs, after which calcium content was determined according to the above-mentioned procedure and was expressed as μg/mg of wet aortic tissues.

The formation of a mineralized matrix was determined by Alizarin Red S staining. HASMCs were fixed in 70% ethanol for 1 h at room temperature and stained with 40 mM Alizarin Red S for 10 min. Then, cells were washed with PBS to eliminate nonspecific staining and the stained matrix was photographed using a digital microscope; Red-stained cells were manual counted and the percentage of Ca-positive cells was calculated.

### ALP activity

ALP activity was measured using a p-nitrophenyl substrate supplied with an ALP Assay Kit (BioChain, Hayward, CA, USA). ALP activity was normalized according to the protein concentration [33].

### Extraction of cellular proteins

Nuclear protein extracts were prepared as previously described [31]. In brief, after being washed with ice-cold PBS, cells were scraped off of the plates with a cell scraper in 1 mL of ice-cold buffer A (10 mM HEPES/NaOH, pH 7.9, 10 mM KCl, 1.5 mM MgCl_2_, 1 mM dithiothreitol [DTT], 0.5 mM PMSF, 2 μg/mL aprotinin, 2 μg/mL pepstatin, and 2 μg/mL leupeptin). After centrifugation at 500x*g* for 10 min at 4°C, the cells were resuspended in 80 μl of buffer B (buffer A containing 0.1% Triton X-100) by gentle pipetting. The cell lysates were allowed to stand on ice for 10 min and then centrifuged at 12,000x*g* for 10 min at 4°C. Nuclear pellets were re-suspended in 70 μl of ice-cold buffer C (20 mM HEPES/NaOH, pH 7.9; 1.5 mM MgCl_2_; 1 mM DTT; 0.2 mM EDTA; 420 mM NaCl; 25% glycerol; 0.5 mM PMSF; 2 μg/mL aprotinin; 2 μg/mL pepstatin; and 2 μg/mL leupeptin), incubated on ice for 30 min with intermittent mixing, and then centrifuged at 15,000x*g* for 30 min at 4°C.

Cell membrane fractions were prepared as described previously [34], with modifications. Briefly, HASMCs were lysed in lysis buffer (10 mM Tris-HCl, 1 mM EDTA, 1 mM PMSF, 10 μg/mL aprotinin, and 0.5 μg/mL leupeptin, pH 7.5). The cell lysates were centrifuged at 3000xg for 20 min. Pellets were re-suspended in lysis buffer and designated as the membrane fraction. Total cell lysates were prepared in lysis buffer (20 mM Tris-HCl, 150 mM NaCl, 1 mM EDTA, 1mM EGTA, 1% Triton, 2.5 mM sodium pyrophosphate, 1 mM β-glycerophosphate, 1 mM Na_3_VO_4_, 1 μg/mL leupeptin, and 1 mM PMSF, pH 7.5). The protein concentrations were determined with BioRad Protein Assay Reagent (Bio-Rad, Hercules, CA), and the samples were stored at -70°C.

### Western blot analysis

Western blot analysis was used to determine the changes in the cell-surface levels of the NADPH oxidase component p47^phox^, the translocation of cytosolic NF-kB p65 to the nucleus, as well as the level of MSX-2, a transcription factor in the nucleus of HASMCs stimulated with TNF-α. Proteins were separated by sodium dodecyl sulfate polyacrylamide gel electrophoresis (SDS-PAGE) and transferred to polyvinylidene fluoride (PVDF) membranes (Millipore, Bedford, MA). The membranes were blocked with 5% milk and then probed with anti-p47^phox^, anti-TR1, anti-p65, goat anti-MSX-2, BMP-2, and RUNX2 (1:1000) antibodies. Then, they were incubated with horseradish peroxidase (HRP)-conjugated secondary antibodies. The proteins were visualized with an enhanced chemiluminescence detection kit (Amersham Biosciences, Piscataway, NJ). Anti-β-actin (1: 5000), anti-caveolin-1 (1:1000) and anti-hnRNA c1/c2 (1:1000) antibodies were used as loading controls. Protein expression levels were quantified as optical densities using ImageQuant software v. 5.2.

### NADPH oxidase (Nox) activity assay and hydrogen peroxide determination

NADPH oxidase activity was determined with superoxide-dependent lucigenin chemiluminescence, as previously described [35]. Cell membrane extract (40 μg) and 5 μM of dark-adapted lucigenin were added to a 96-well luminometer plate and adjusted to a final volume of 250 μl with oxidase assay buffer before 100 μM of NADPH was added. Relative light units (RLUs) were read with a luminometer (Dynatech ML2250, Dynatech Laboratories Inc., VA). Light emission was recorded every minute for 15 min and expressed as mean RLUs/min.

The effect of antioxidant reagents on ROS production in HASMCs was determined by a fluorometric assay using DCFH-DA as the probe. This method is based on the oxidation by H_2_O_2_ of nonfluorescent DCFH-DA to fluorescent 2’,7’- dichlorofluorescin (DCF). Confluent HASMCs in 24-well plates will be pretreated with various concentrations of antioxidant reagents. The cells were washed with PBS, and then serum-free M231 containing 10 μM DCFH-DA was added and incubation continued for 45 min at 37°C. The fluorescence intensity (relative fluorescence units) was measured at 485 nm excitation and 530 nm emission using a Fluorescence Microplate Reader.

### siRNA transfection

Small interfering RNA (siRNA) oligonucleotides against TNFR1, p65, and p47 were suspended at a concentration of 10 μM. Cells were seeded for transfection in medium without antibiotics at one day before transfection to ensure that they were 85%–95% confluent on the day of transfection. For transfection, regular medium was replaced with serum-free medium without antibiotics. The cells were transfected with siRNA using oligofectamine at a ratio of 1 siRNA: 2 oligofectamine (μg:μl) at a final concentration of 25–50 nM siRNA. The cells were incubated with the siRNA-oligofectamine complex for 5 h, the serum-free medium was replaced with normal medium (containing 10% FBS) without antibiotics, and the cells were incubated for a total of 48 h before further analysis.

### Statistical analysis

All data were expressed as the mean±SEM for the continuous variables and as the number (percent) for the categorical variables. Intergroup comparisons were performed by Student's *t* test or one-way ANOVA. A p<0.05 was considered statistically significant. SPSS 9.0 (version 12, SPSS, Chicago, Illinois, USA) software package was used for all statistical analyses.

## Results

### Effects of simvastatin (SIM), on the high-fat diet (HFD)-induced macrophage infiltration and the aortic calcification in the aortic medial layer in LDLR -/- mice

**Fig 1:**
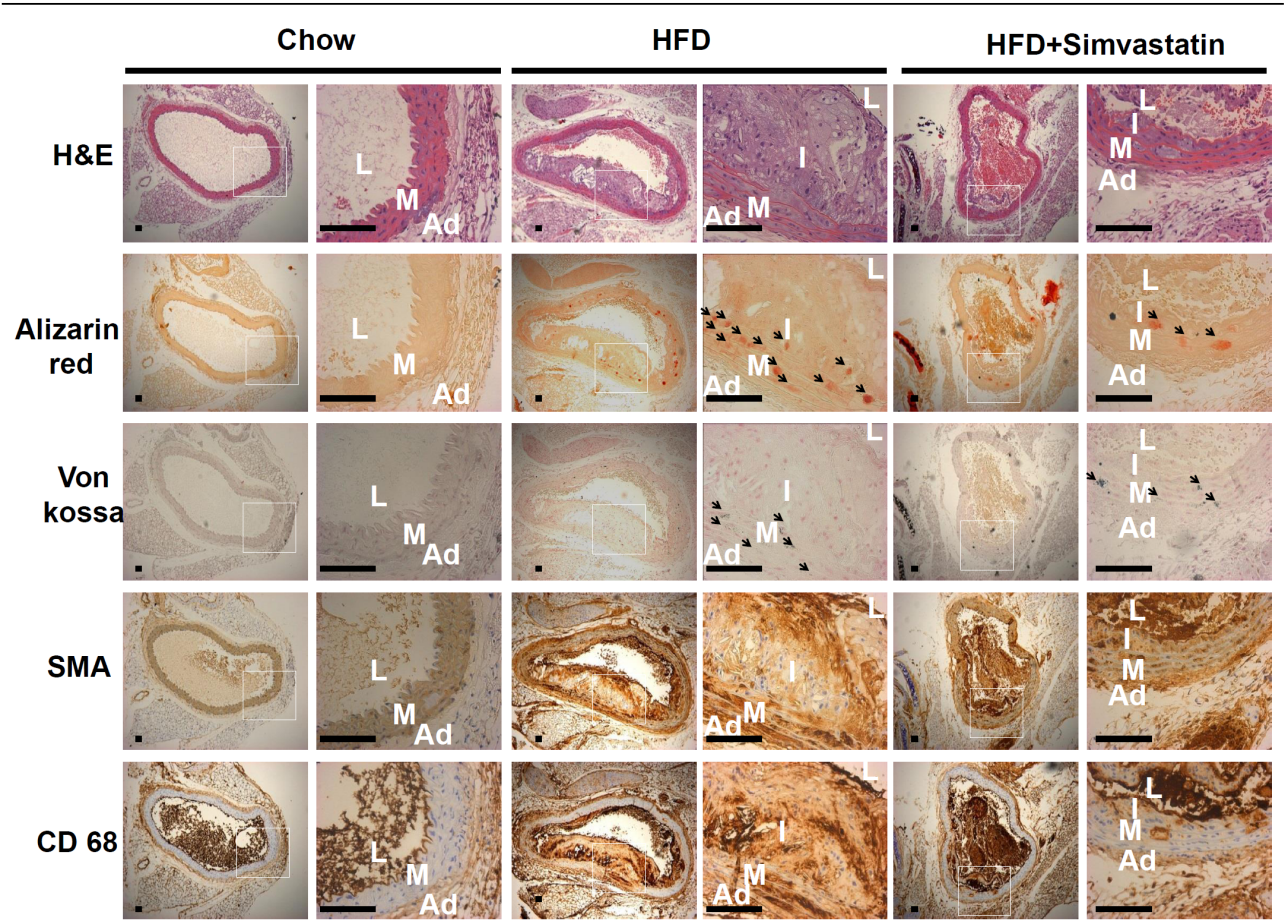
HFD promoted calcification, which was attenuated by SIM, in male LDLR-/- mice. Mice were fed a chow diet and high-fat diet (HFD) for 6 months. The mice aorta tissue was isolation cut from heart to diaphragm and separated 4 parts of thoracic aorta section. Close to the heart of the section can be observed significant arterial calcification. HFD-induced artery atherosclerotic plaque formation was detected by H&E staining. HFD-induced artery calcification was detected by alizarin red and van kosa staining. α-SMA, was used as a smooth muscle cell marker, CD68 staining demonstrated infiltration of macrophages in the media. HFD promoted aortic calcification, which was dominant in the media layer. The arrows point to the area of calcium deposition. In the lumen have blood cell remnants. L: lumen, I: intima, M: media, and A: adventitia. (ND = 6, HFD = 10, and HFD+SIM = 10). LDLR-/- mice were photographed at 100x magnification. Scale bars represent 75 μm. LDLR-/- mice were photographed at 400x magnification. Scale bars represent 15 μm.

LDLR-/- mice were fed a normal diet (n = 6) or an HFD (n = 10) for 6 months. As predicted, HFD-induced artery atherosclerotic plaque formation was detected by H&E staining (Fig 1). It appears that the atherosclerotic lesion in the section of the aorta next to the heart appears to be more severe than the other 3 sections. Alizarin red staining indicates HFD induced calcification is more prevalent in the medial layer of the proximal aorta. SIM appears to attenuate medial calcification. HFD appears to trigger the infiltration of activated macrophage into the medial layer as indicated by the CD68 staining (Fig 1). SIM treatment significantly reduced the activation and infiltration of macrophages into the medial layer.

### Effects of simvastatin (SIM), N-acetyl cysteine (NAC) and apocynin (APO) on the high-fat diet (HFD)-induced aortic calcification and the serum 3-nitro-tyrosine levels in LDLR -/- mice

**Fig 2:**
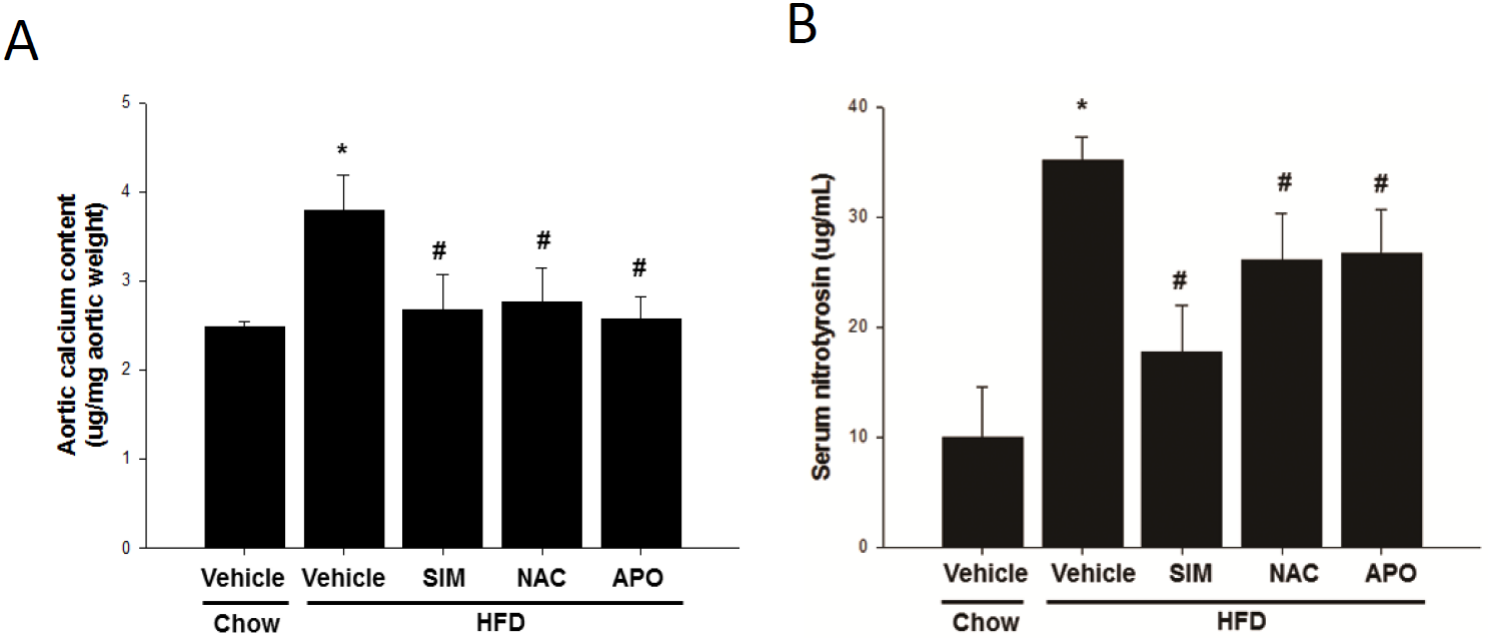
Stimulation of calcification deposition and serum nitrotyrosine formation by HFD was attenuated by SIM, NAC and APO in male LDLR-/- mice. Mice were fed a chow diet or high-fat diet (HFD) simultaneously treated with SIM, NAC or APO for 6 months. (A) Aortic calcium levels. (B). Serum oxidative marker levels nitrotyrosine were assayed. *p<0.05 compared to the control group, and #*p*<0.05 compared to the TNF-α groups (ND = 6 and HFD = 10, SIM = 15, NAC = 5, and APO = 5).

Fig 2A showed that HFD increased the acid-extractable calcium deposition in the aortic matrix from 2.49+0.25 (n = 6) to 3.79+1.13 μg Ca/mg aortic tissue (n = 10). This HFD induced aortic Ca deposit is significantly reduced by SIM (n = 15), NAC (n = 5) or APO (n = 5) treatments.

Fig 2B showed the effect of HFD and SIM, NAC and APOS on serum 3-nitro-tyrosine. It has been known that oxidative stress increases the production of superoxide (O2−) and NO forming peroxynitrite (ONOO−) which is a destructive free radical oxidant capable of oxidizing several lipoproteins and nitrating tyrosine residues in many proteins. It is difficult to measure the production of ONOO− since ONOO is very unstable. Thus, nitrotyrosine is used as a surrogate for ONOO− [36]. HFD induced oxidative stress as indicated by the elevation of serum 3-nitro-tyrosine (35.1±2.1 versus 9.9±4.6 mg/dl). SIM, APO or NAC partially block the HFD induced oxidative stress as indicated by the lowered serum nitro-tyrosine).

HFD induced dyslipidemia and diabetic condition and dyslipidemia and diabetic condition which are believed to contribute to aortic calcification (Table 1). SIM, APO, NAC did not lower cholesterol, plasma triglycerides or glucose level yet they are all effectively retarded HFD induced aortic calcification (Fig 2A). Apparently, the protective effect of SIM against aortic calcification is not contributed by the SIM cholesterol lowering effect. Since APO and NAC are anti-oxidant reagents, thus, we suspect the protective effect of SIM may, in part, contributed by its in vivo anti-oxidant activity.

**Table 1:** Metabolic parameters after 6 months of dietary challenge and simvastatin (10 mg^-1^kg^-1^day^-1^), NAC (250 mg^-1^kg^-1^day^-1^), or apocynin (40 mg^-1^kg^-1^day^-1^) treatment in LDLR-/- mice.

Parameters list	Chow (n = 6)	HFD (n = 10)	HFD + Simvastatin(n = 10)	HFD + NAC (n = 5)	HFD + Apocynin (n = 5)
Calcium, (mg/dL)	8.9±0.2	9.1±0.2	9.1±0.1	8.9±0.1	8.8±0.5
ALP, (u/L)	66±10	85±11^a^	83±20	74±15	84±13
PHOS, (mg/dL)	8.4±0.6	7.1±0.6	8.2±1	8.5±1.7	8.2±0.4
Glucose, (mg/dL)	104±8	225±39^a^	223±32	262±38	258±30
CHOL, (mg/dL)	272±29	1377±153^a^	1390±241	1292±208	1239±272
HDL, (mg/dL)	50.7±16.5	70±2.9 ^a^	66.5±5.1	67±5.2	64±7.1
LDL, (mg/dL)	107 ±12	659±139 ^a^	660±154	585±171	605±131
TGs, (mg/dL)	123±17	614±154^a^	660±133	558±192	594±197
TNF-α, (pg/mL)	2.8±0.6	8.1±1.6 ^a^	5.4±1.4 ^b^	5.7±2.7^b^	5.2±2.2 ^b^
Weight, (g)	27.2±2.2	40.3±3.9^a^	36.1±6.1	35.4±5.8	39.6±5.7

Values are the mean ± standard deviation (SD).

^a^P < 0.05 for chow versus HFD,

^b^P < 0.05 for HFD versus HFD+simvastatin, HFD+NAC or HFD+Apocynin.

LP: Alkaline phosphatase; PHOS:Phosphate; CHOL: Cholesterol; HDL: High-density lipoprotein cholesterol; LDL: Low-density lipoprotein cholesterol; and TGs: Triglycerides; TNF-α: Tumor necrosis factor alpha.

### Effects of HFD, SIM, APO, or NAC on the metabolic parameters of wild type and LDLR -/- mice

Tables 1 and 2 showed the impact of HFD on LDLR -/- and wild type metabolic parameters. HFD caused the WT and LDLR -/- animals to develop dyslipidemia and glucose intolerance. The severities of the dyslipidemia and glucose intolerance are much lower for the wild type (Tables 1 and 2). HFD induced a significant increase in alkaline phosphatase activity and TNF-α in both the wild type (WT) and LDLR -/- animals. Alkaline phosphatase (ALP) is a by-product of osteoblast activity and it is used as a molecular marker for vascular calcification [19]. TNF-α has been found to play a crucial role in the calcification of vascular smooth muscle cells *in vitro* and *in vivo* [16, 17]. The elevation of ALP, TNF-α, serum glucose and dyslipidemia lead us to suspect that HFD is able to induce aortic calcification for the WT. Since HFD induced dyslipidemia and diabetic is much less severe for the WT, we suspect that it may take a lot longer HFD feeding to induce WT to develop significant aortic calcification.

**Table 2:** Metabolic parameters after 20 weeks of dietary in wild type C57/B6J mice.

Parameters list	Chow (N = 6)	HFD (N = 6)
Glucose, (mg/dL)	87±12	155±17^a^
CHOL, (mg/dL)	124±49	316+44^a^
HDL, (mg/dL)	42±20	114+12^a^
LDL, (mg/dL)	7.5±3	36+10^a^
TG, (mg/dL)	153±87	139+26
BUN, (mg/dL)	26.4±8	27.4±4.2
CREA, (mg/dL)	0.6±0.2	0.5±0.2
TBIL, (mg/dL)	0.03±0.01	0.11±0.02
ALP, (u/L)	60±19	130±44^a^
ALT, (mg/dL)	32±19	241±136^a^
TNF-α, (pg/mL)	3.6±2	10±3^a^

Values are mean ± standard deviation (SD).

^a^P < 0.05, for chow versus HFD.

CHOL: Cholesterol; HDL: High-density lipoprotein cholesterol; LDL: Low-density lipoprotein cholesterol; TG: Triglyceride; BUN: Blood urea nitrogen; CREA: Creatinine; TBIL: Total bilirubin. ALP: alkaline phosphatase; ALT: The alanine aminotransferase; TNF-α: Tumor necrosis factor alpha.

HFD drastically increased the serum TNF-α level in the LDLR -/- mice and SIM, NAC and APO treatment effectively attenuate HFD fed LDLR-/- mice pro-inflammatory cytokine expression (Table 1) and oxidative stress as indicated by 3-nitro-tyrosine (Fig 2B). We were expecting treatments retarding calcification to lower the HFD fed animal ALP, but we did not.

We hypothesized that TNF-α activation lead to ROS production and the increase in ROS stimulates activate MSX2 program to cause a pro-calcific response. This hypothesis explains why antioxidant reagents like NAC and APO would attenuate HFD induced oxidation stress, elevation of TNFR1 and aortic calcification.

### Effect of and SIM on HASMC calcium deposition and bone markers expression

**Fig 3:**
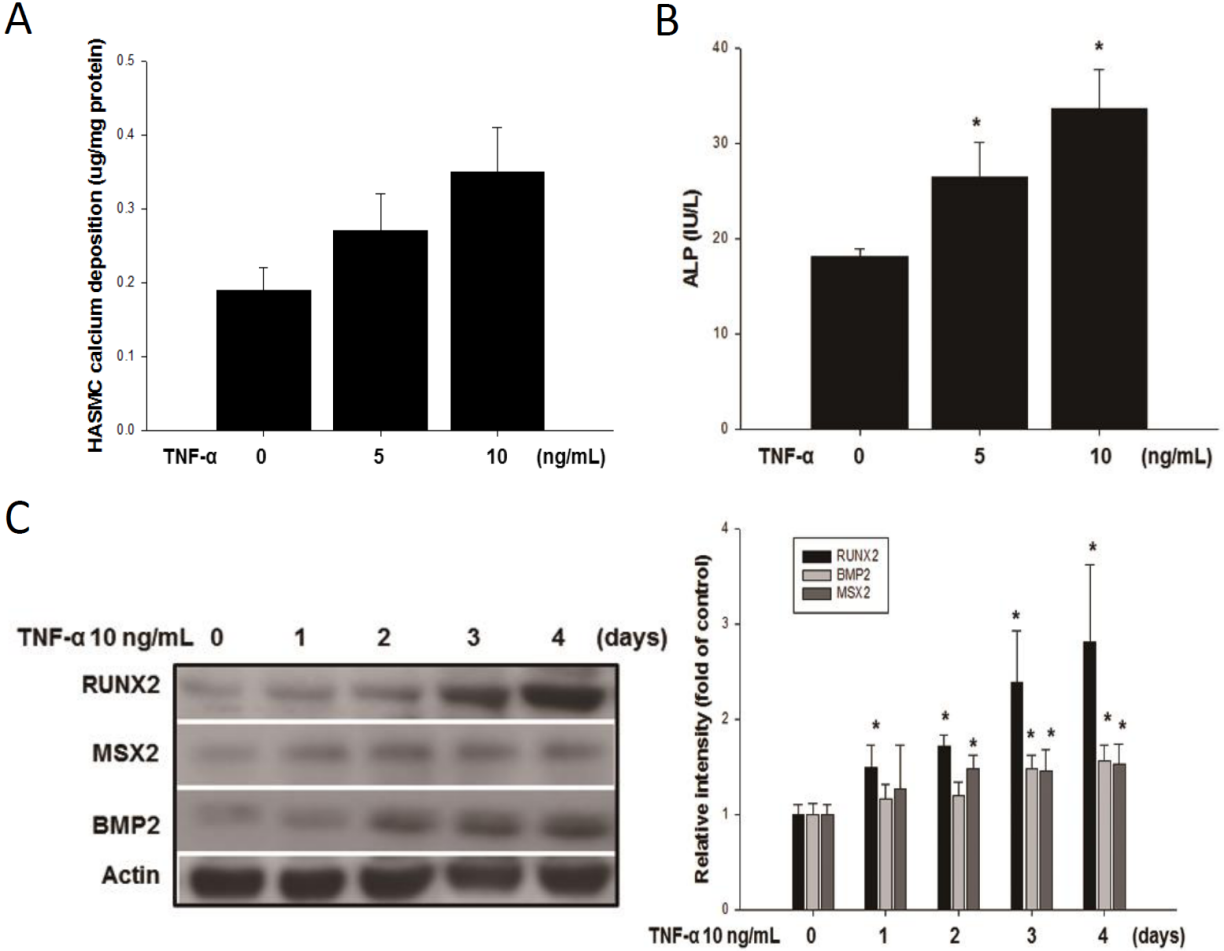
HASMCs were cultured in osteogenic differentiation medium for 4 days in presence or absence of TNF-α. The calcium deposition (A) and ALP activity (B) were induced dose-dependently by TNF-α for 4 days. Whole cell lysate western blotting results showed osteogenic markers of MSX-2, BMP2, and RUNX2 protein accumulation (C) was increased by TNF-α (10 ng/mL) for the indicated time in the HASMCs. N = 6 for each set of experiments. *p<0.05 compared to the control group.

Our working hypothesis postulates that TNF-α stimulates HASMCs differentiation into osteoblast type of cell linage to induce calcium deposition. If this working hypothesis is true, then treating HASMC with TNF-α should result in increases HASMC calcium deposition, increases in osteogenic marker proteins expression and ALP activity. HASMC were cultured in osteogenic differentiation medium in the presence or absence of non-toxic concentrations of TNF-α (5 and 10 ng/mL) for 4 days. We found that TNF-α induced HASMC calcium deposition, upregulate ALP activity and increase in BMP2, MSX2, and RUNX2 expression (Fig 3A, 3B and 3C). Data are in agreement with our speculations.

**Fig 4:**
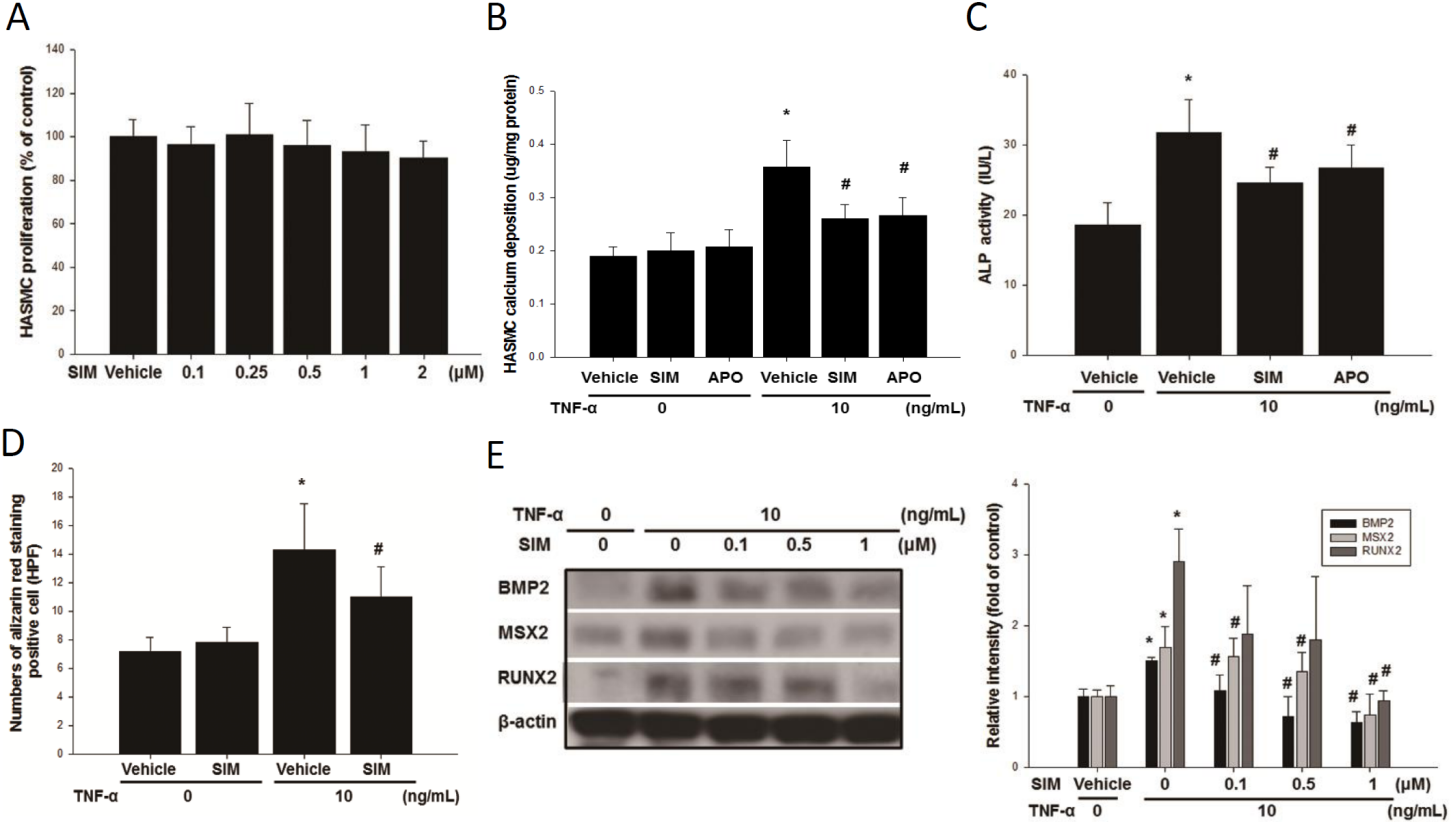
The effect of SIM attenuated TNF-α-induced calcium deposition in HASMCs. The cytotoxicity effect of SIM (A) was assayed by MTT. The induction of calcium deposition (B) and ALP activity (C) by TNF-α in HASMCs was attenuated by SIM and APO. SIM treatments attenuated the TNF-α-induced number of alizarin red stained cells (D). Induction of MSX-2, BMP2, and RUNX2 accumulation by TNF-α was blocked by SIM (E). N = 6 for each set of experiments. *p<0.05 compared to the control group, and #*p*<0.05 compared to the TNF-α groups.

We culture HASMCs in an osteogenic differentiation medium in the presence or absence of non-toxic concentrations of TNF-α (5 and 10 ng/mL) for 3 days, followed by SIM (1 μM) treatment for 1 day. Treating HASMC with 2 μM of SIM for 24 hours did not produce any adverse effect on cell vitality as indicated by MTT assay (Fig 4A). If SIM and APO reduce HFD induced aortic calcification by reducing TNFR1 expression to lower ROS production and the artery calcification. Therefore, SIM and APO should significantly reduce TNF-α-induced calcium deposition in HASMC. In addition, SIM and APO treatments should also lower the TNF-α-induced ALP activity, reduce number of Alizarin red stained cells, and down regulate the osteomarker proteins expression. Experimental data are in agreement with our speculation. SIM and APO treatment significantly reduced the TNF treated HASMC calcium deposition, number of Alizarin stained cells, ALP activity, and BMP2, MSX2, and RUNX2 expression (Fig 4B, 4C, 4D and 4E).

### TNF-α-induced oxidative stress was reduced by APO and SIM in HASMCs

**Fig 5:**
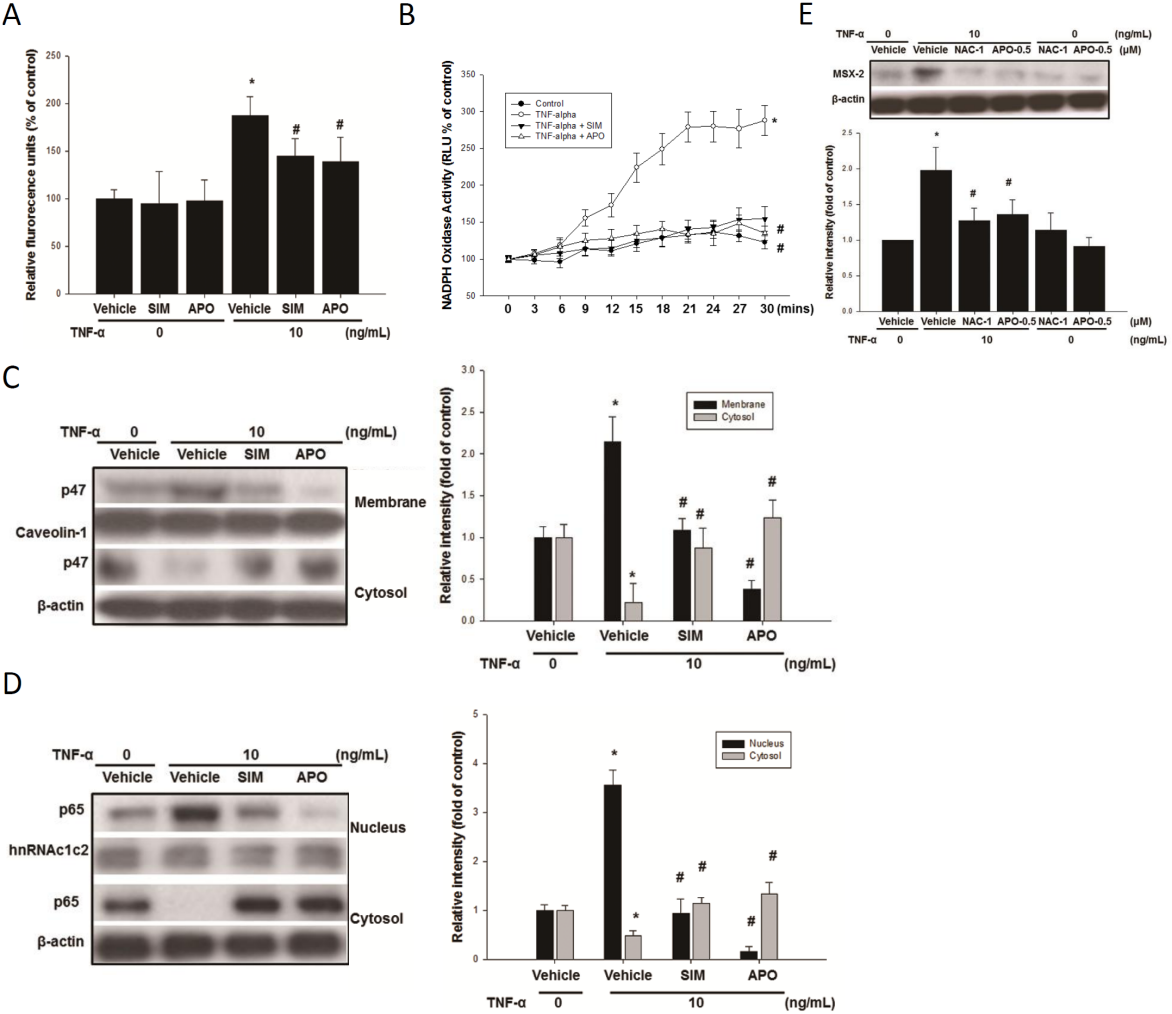
Anti-oxidative activity is involved in suppression of TNF-α-mediated p47 and p65 translocation by SIM in HASMCs. HASMCs were cultured in osteogenic differentiation medium for 1 day in the presence or absence of TNF-α (10 ng/mL) concomitantly with SIM (1 μM) or apocynin (500 μM). Intracellular hydrogen peroxide generation was assessed by DCF-AM staining (A). NADPH oxidase activity was evaluated by lucigenin chemiluminescence (B). The expression levels of the NADPH oxidase subunit p47 (membrane fraction) and NF-κB subunit p65 (nuclear fraction) were assessed by western blotting. Caveoli-1 was used as a membrane fraction loading control, and hnRNAc1/c2 was used as a nuclear fraction loading control (C and D). Antioxidant agents attenuated TNF-α-induced bone marker MSX-2 accumulation in HASMCs (E). *p<0.05 compared to the control group, and #*p*<0.05 compared to the TNF-α groups. N = 6 for each set of experiments.

Our hypothesis postulates that activation of TNFR by TNF-α lead to oxidative stress (ROS production) and ROS promotes VSMC to differentiate into osteogenic cell linage which leads to aortic calcification [10]. According to this hypothesis, one would expect antioxidants capable of neutralizing the ROS should lower the oxidative stress to blunt the expression osteogenic protein markers expression and lower the ALP activity. As predicted by the working hypothesis, treating HASMC with TNF-α increased the NADPH oxidase activity and the level of H_2_O_2_ (Fig 5A and 5B). SIM and APO only reduced the level of oxidative stress by partially offset the production induction of H_2_O_2_ by TNF-α yet both treatments significantly abolish the effect of TNF-α induced elevation of NADPH activity (Fig 5A and 5B). These results suggest a possibility that the protective effect of SIM and APO against aortic calcification is, in part, contributed by their ability to block activation of NADPH oxidase. Similar result also could find in previously study [37]. As found with LDLR-/- model, NAC is as effective as APO in attenuating the TNF-α-induced MSX-2, RUNX2 and BMP2 accumulation in HASMCs (Fig 5E and S1 Fig).

### TNF-α-induced translocation of p47 phox was reduced by APO and SIM in HASMCs

NADPH oxidase consists of membrane-bound (gp91phox and p22 phox) and cytosolic components (p47 phox, p67 phox, p40 phox, and Rac proteins). NADPH oxidase activation requires the translocation of the cytosolic components p47 phox to cell membrane [38]. Western blotting showed that TNF-α induced the translocation of cytosolic p47 to the cell membrane in the HASMCs, and caveolin-1, which is a membrane protein, was used as a loading control. SIM and APO significantly attenuated p47 translocation. (Fig 5C)

### TNF-α-induced translocation of NF-κB was reduced by APO and SIM in HASMCs

It is known that TNF-α can induce MSX2 expression via the NF-κB pathway [39]. P65 is a surrogate for NF-κB activation under TNF-α-stimulated condition. Therefore, we analyzed NF-κB subunit p65 translocation to nucleus in this study and found that SIM significantly inhibited TNF-α-mediated cytosolic p65 translocation to the nucleus. Interestingly, similar results also could find in APO treatment (Fig 5D).

### SIM attenuated the expression of TNFR1 in HASMCs

**Fig 6:**
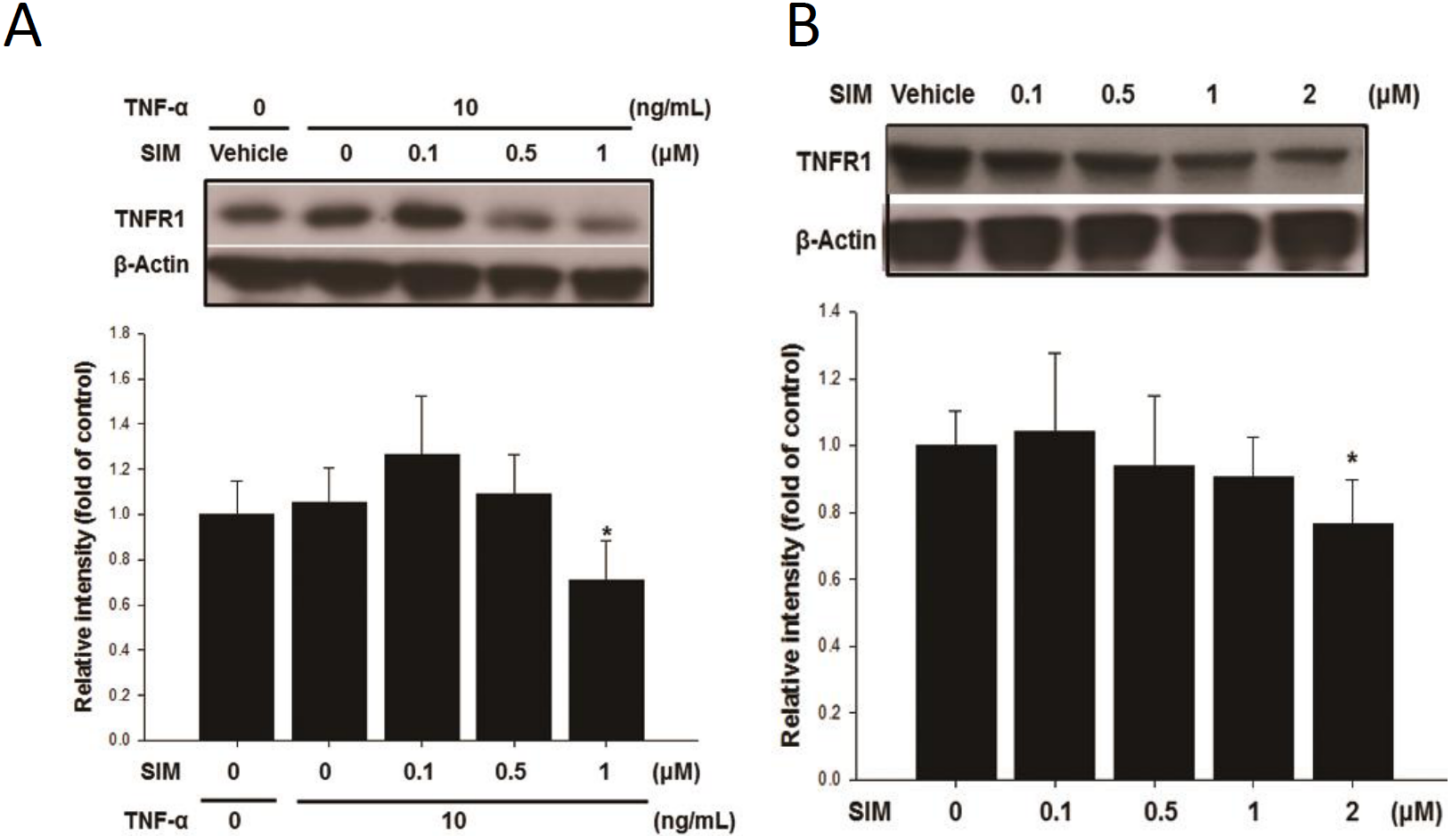
SIM attenuated TNF-α-mediated TNFR1 accumulation in HASMCs. Western blotting assay (A) revealed that 1 uM of SIM could decrease TNFR1 expression in the absence of TNF-α (A), while 2 uM (B) had significant effects on its expression in the presence of TNF-α. *p<0.05 compared to the control group. N = 6 for each set of experiments.

It is known TNFR1 plays a crucial role in TNF-α-induced ROS generation [16] and in BMP-2-induced osteoblastic differentiation in C2C12 cells [40]. Therefore, we investigated the possible role of TNFR1 in the inhibition of the effect of TNF-α by SIM. TNF-α treatment stimulates TNFR1 accumulation and, 1 μM of SIM could significantly suppress the expression of TNFR1 (Fig 6A). I look even 2 μM of SIM to produce a small reducing the TNFR1 expression of the untreated cells (Fig 6B).

### siRNAs against TNFR1, p65 and p47 attenuated the expression of TNF-α-induced osteogenic markers in HASMCs

**Fig 7:**
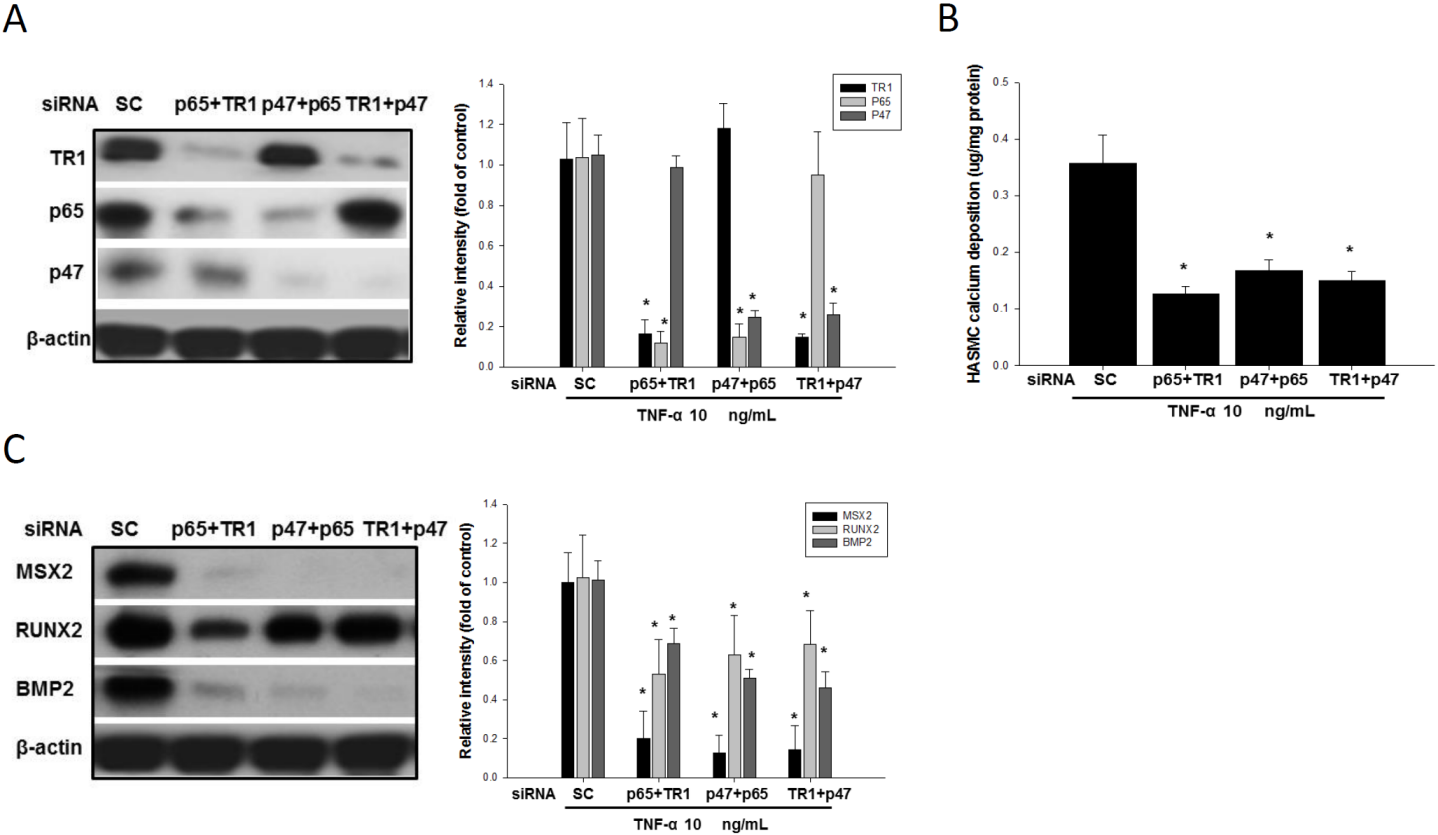
siRNAs against TNFR1, p65 and p47 down regulated calcium deposition and bone marker expression. Western blotting assay the effects of the knock-down of the TNFR1, p65 and p47 proteins by these siRNAs (A) indicated that they were functioning. Compared with the TNF-α-stimulated cells in the presence of scrambled siRNAs, any combination of two of the TNFR1, p65 or p47 siRNAs, dramatically abolished TNF-α-stimulated calcification (B) and bone marker expression in the HASMCs (C). *p<0.05 compared to the control group. N = 6 for each set of experiments.

We further investigated the effects of the combined use of siRNAs against TNFR1, p65 and p47 on the expression of osteogenic markers because the use of a single siRNA did not have significant effects (S2 Fig). The knockdown approach showed that the combination of TNFR1, p65, and p47 siRNAs are effective in reducing the expression of these factors, calcium deposition of and the expression of BMP-2, Msx2, and Runx2 (Fig 7A, 7B and 7C), The single siRNA treatment failed to effectively down-regulates the TNF-alpha induced bone markers (S2 Fig). However, treating the HASMC with a mixture of two siRNA significantly lowers the TNF-alpha induced bone markers (Fig 7C). These findings lead us to believe that the synergistic interactions among TNFR1, p65, and p47 play important roles in TNF-alpha induced bone markers synthesis.

### Effect of SIM on TNFR1 in HFD-fed LDLR -/- mice

**Fig 8:**
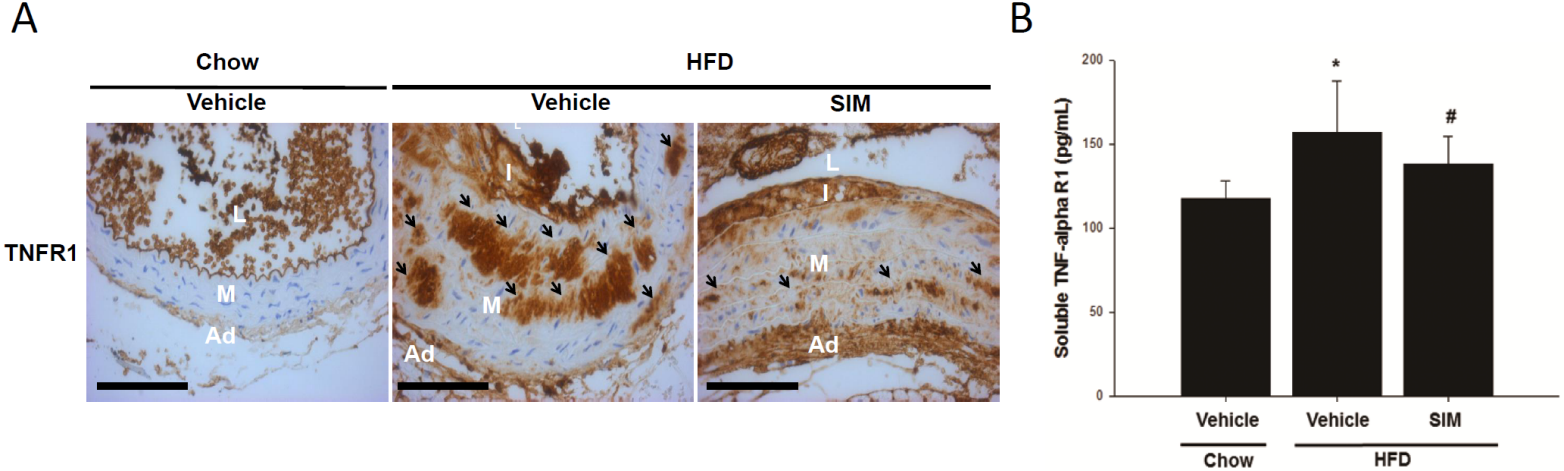
Expression of TNFR1 was induced by HFD and could be attenuated by SIM in male LDLR-/- mice. TNFR1 protein accumulation in close to the heart of the section of thoracic aorta was determinate by IHC staining. The immunostaining of aortic TNFR1 (A) showed that SIM could significantly decrease the stimulatory effects of the HFD on TNFR1 and ELISA assay of the plasma level of sTNFR1 (B) and L: lumen, I: intimal, M: media, and A: adventia. *p<0.05 compared to the control group, and #*p*<0.05 compared to the TNF-α groups. (ND = 6, HFD = 10, SIM = 15)

Finally, we confirmed the effect of SIM on TNFR1 accumulation in the media layer of the aorta of the HFD-fed mice by IHC staining. The results showed that TNFR1 was significantly increased in the HFD-fed mice and could be attenuated by SIM (Fig 8A). We next assessed the concentration of sTNFR1 in circulation. The results showed that compared with the chow diet, the HFD increased the serum sTNFR1 level, which could be decreased by SIM (chow diet, 117.6±10.6; HFD, 156.9±30.5; and HFD+SIM, 138.0±16.5 pg/mL) (Fig 8B).

## Discussion

There are several types of vascular calcification, such as atherosclerosis and medial calcification, which are often observed in elderly people and patients with diabetes mellitus and/or chronic kidney disease [1, 17]. In the present study, medial calcification predominated in HFD-fed LDLR-/- mice. HFD up-regulate TNF-α, oxidative stress, BMP2, MSX2, RUNX2, p47, p65 and TNFR1 accumulation in both the HFD-fed LDLR-/- mice and the TNF-α-treated HASMC system.

APO, NAC or SIM reduced the HFD-induced elevation of serum TNF-α (Table 1) and nitrotyrosine (Fig 2B) and calcium deposition in the aorta (Fig 2A). However, APO, NAC, or SIM produced no effects on the HFD-induced increases in serum glucose, cholesterol, HDL, LDL, or triglyceride levels or body weight (Table 1). Since both APO and NAC lowered the HFD-induced TNF-αsynthesis, aortic calcium deposition, and nitrotyrosine formation, we decided to only assess APO activity in the HASMC model.

APO inhibited the TNF-α induced translocation of cytosolic p47to the cell membrane (Fig 5C) to prevent the activation of cell membrane bound NADPH oxidase (fig 5B). APO produced a real but reactively small impact on was very effect on TNF-α-induced H_2_O_2_ production NADPH oxidase activity (Fig 5A). These results indicate that NADPH oxidase activation plays a major role in the TNF-α induced oxidative stress. As with APO, SIM (1 μM) effectively block the activation of NADPH oxidase yet produced a real but reactively small impact on the TNF-α induced H_2_O_2_ production (Fig 5A, 5B and 5C).

It is known that oxidative stress can result in aortic valve (AV) calcification [41]. In preclinical models of diabetic arterial diseases, TNFα-dependent NADPH oxidase activation [42] has been shown to promote Runx2/Cbfa1 expression and the osteogenic mineralization of vascular smooth muscle [5]. In the present study, we demonstrated that SIM and APO blocked that TNF-α induced NADPH oxidase activation stimulated BMP2, MSX2, and RUNX2 accumulation and calcium deposition in HASMCs. APO prevents the translocation of p47 into cell membrane to block the activation of NADPH oxidase. We suspect SIM blocks the activation of NADPH oxidase by the same mechanism. Previous evidence has shown that in endothelial cells, both TNF-α and cell stretching promote the NADPH oxidase–mediated production of H_2_O_2_, which leads to the nuclear translocation of NF-κB [43, 44]. NF-κB activation is involved in ICAM-1 up-regulation, suggesting that NADPH oxidase is required by TNF-α for the sequential phosphorylation of NF-κB in HUVECs [45]. Furthermore, BMP-2, BMP-4, RUNX-2, and MSX-2 expression can be regulated by the TNF-α-mediated activation of NF-κB [39, 44, 46, 47].

Statin inhibit hepatic cholesterol synthesis. In response to the lowered cholesterol synthesis, liver expresses more LDLR to increase LDL uptake to source cholesterol for bile synthesis. As a result, level of LDL in circulation is reduced [48]. The animal we used does not express LDLR (LDLR-/- mice), thus, statin cannot enhance the LDL uptake to lower cholesterol. SIM has been reported to reduce atherosclerotic lesions in hypercholesterolemic mice without reducing lipid levels [49]. SIM was also reported to mitigate inflammation and thrombogenicity in hypercholesterolemic pigs and monkeys without affecting cholesterol levels [50, 51]. In our present study, SIM didn’t affect the cholesterol yet is still functional in lowering HFD-induced aortic calcification as well. We thus assessed the effects of SIM and APO on NF-κB in HASMCs. We found that SIM and APO inhibited the TNF-α induced translocation of the NF-κB subunit p65 to the nucleus in the HASMCs (Fig 5D). These finding suggested that SIM mitigating calcification blocking MSX2 accumulation. TNF-α produced by activated macrophages or fat cells transmits signals through two distinct surface receptors, TNFR1 and TNFR2 [52]. In our *in vivo*study, SIM attenuated the HFD induced elevation of serum TNF-α (Table 1) and sTNFR1 (Fig 7A), and the TNFR1 in the aorta (Fig 7B). SIM also decreased the expression of TNFR1 in the TNF-α treated HASMCs. These results suggest that lowering the HFD induced TNFR1 accumulation and blocking the activation of NADPH oxidase both contributes to the protective effect against aortic calcification.

Combinations of p47, p65 or TNFR1 siRNAs mitigated the osteogenic effects of TNF-α in the HASMCs (Fig 6C and 6D). HFD was found to up-regulate LDLR-/- mice TNFR1 accumulation and SIM was effective in mitigate the HFD effect on TNFR1 accumulation (Fig 7A). Our findings suggested that TNF-α and oxidative stress trigger aortic calcification.

Our results are in agreement with some previous studies reporting that TNF-α stimulates the mineralization of aortic calcifying vascular cells *in vitro* [53], and infliximab, which is an antibody against TNF-α, can inhibit its signaling in LDLR-/- mice to down-regulate osteogenic factors [16]. Further, our results support previous studies indicating that antioxidants, such as tempol, are potentially useful to prevent the progression of vascular calcification in CKD [54] and that α-lipoic acid attenuates vascular calcification via the reversal of mitochondrial function and restoration of the Gas6/Axl/Akt survival pathway [55]. Our findings are also in line with previous studies reporting that TNF-α induces MSX2 and procalcific responses in myofibroblasts via TNFR1 [18] and that SIM antagonizes TNF-α to inhibit the induction of osteoblastic differentiation by BMP-2 in C2C12 cells [40].

It is well known that WT mice on HFD typically do not develop noticeable aorta calcification in 6 months while LDLR -/- on HFD developed aortic calcification as indicated by alizarin red staining in as short as 1 months [16]. This discrepancy raised a question if the mechanism causing aortic calcification can be applied to WT animal. We investigate the impact of HFD on the metabolic parameters of WT mice in a preliminary study (table 2). We discovered that HFD caused the WT to develop dyslipidemia and glucose intolerance as it did to LDLR-/-. However, the dyslipidemia and glucose intolerance in the WT are much less severe. Since dyslipidemia and diabetic are shown to correlate with severity of aortic calcification, we suspect that the mechanism causing aortic calcification in LDLR-/- mice may be applicable to the WT. Therefore, we suspect, it may take a much longer feeding for us to detect the protective effect of SIM on WT aortic calcification.

The effects of statins on calcification remain controversial. Some *in vitro* studies have indicated that atorvastatin increases calcium deposition in VSMCs growing in calcification medium [56]. In addition, previous studies have reported that statins can inhibit the calcification of atherosclerotic plaques in ApoE-/- mice [57, 58]. It was reported that atorvastatin therapy did not halt the progression of calcific aortic stenosis in dyslipidemia patients [26]. However, there is contradictory reporting of the beneficial clinical effects of statin medication on calcific aortic stenosis [25, 59]. There are only a few human clinical studies and the results are contradictory, we believe better understanding the mechanism of statin on calcification may help understand the human clinical finding better. We decided to use simvastatin to study the effect of statin on HFD induced aortic smooth muscle calcification because we suspect arresting aortic smooth muscle calcification may benefit healthy aging. We decide not to use atorvastatin because our concerns about the potential negative effect on glucose control [60]. Previously study reported that atorvastatin (80 mg dose) may increase the risk of new onset diabetes (NOD). Although the difference did not reach statistically significant level, there is a trend that 80 mg of atorvastatin increase the risk of NOD more than 10 mg of atorvastatin or 20 mg of simvastatin [60]. Diabetes duration is shown to correlate with severity of aortic media calcification [61].

SIM inhibited the calcification in the HFD fed LDLR-/- mice aorta and TNF-α treated HASMC cell culture. Data strongly suggest that the protective effect of SIM may not be contributed its cholesterol lowering effect via inhibition of HMG-CoA reductase. Instead, the protective effect of SIM against calcification was contributed by 1) blocking the activation of cell membrane surface NADPH, and 2) down regulation of TNF-alpha and TNFR1. The reduction in the inflammation mediators TNF-α and TNFR1 suggest that SIM may suppress the progression of vascular diseases.

Our findings indicate that SIM lowers inflammatory mediator TNF alpha induced TNFR1 to lower the oxidative stress in HASMC and animal models. As a result of this anti-inflammatory effect, SIM retard the progression of artery calcification in LDLR -/- mice. These results provide novel evidence that SIM treatment attenuates arterial calcification and these effects are demonstrated through potent anti-inflammatory and antioxidant properties of SIM. Our findings provide novel evidence that SIM treatment may attenuate arterial calcification. In addition, ample evidence exists in support of the potent anti-inflammatory properties and antioxidant effects of SIM [62]. However, further studies are warranted to confirm these findings and clarify the mechanism of how SIM modulate the pathogenesis of artery calcification.

In summary, in addition to the known roles of statins in influencing endogenous cholesterol levels by inhibiting HMG-CoA reductase and other pleiotropic effects, such as anti-inflammatory, anti-proliferative, and anti-thrombotic effects [63, 64], we demonstrated that SIM behaves like APO, acting via a novel mechanism independent of its blood cholesterol-lowing effect to prevent the membrane translocation of p47phox, thereby suppressing the aforementioned bone markers. In addition, SIM also has direct effects on the expression of the inflammatory cytokine TNF-α and its receptor, TNFR1.

## Supporting Information

S1 Fig APO or NAC suppressed TNF-α-mediated RUNX2 and BMP2 accumulation in HASMCs. HASMCs were cultured in osteogenic differentiation medium for 3 day in the presence or absence of TNF-α (10 ng/mL) concomitantly with apocynin (A) or NAC (B). *p<0.05 compared to the control group, and #*p*<0.05 compared to the TNF-α groups. N = 6 for each set of experiments.
(TIF)

S2 Fig The effect of bone marker expression under siRNAs against TNFR1, p65 and p47. Western blotting assay the effects of the knock-down of the TNFR1, p65 and p47 proteins by these siRNAs indicated that they were functioning. Compared with the TNF-α-stimulated cells in the presence of scrambled siRNAs, any of the TNFR1, p65 or p47 siRNAs, didn’t dramatically abolished TNF-α-stimulated bone marker expression in the HASMCs. N = 6 for each set of experiments.
(TIF)
