# Correction: Simvastatin Attenuates Oxidative Stress, NF-κB Activation, and Artery Calcification in LDLR-/- Mice Fed with High Fat Diet via Down-regulation of Tumor Necrosis Factor-α and TNF Receptor 1

**DOI:** 10.1371/journal.pone.0148590

**Published:** 2016-01-29

**Authors:** Chih-Pei Lin, Po-Hsun Huang, Chung Fang Lai, Jaw-Wen Chen, Shing-Jong Lin, Jia-Shiong Chen

There is an error in affiliation 2 for author Chih-Pei Lin. Affiliation 2 should be: Department of Biotechnology and Laboratory Science in Medicine and Institute of Biotechnology in Medicine, National Yang-Ming University, Taipei, Taiwan.
