# Supplementary figures and images for: Simvastatin Attenuates Oxidative Stress, NF-κB Activation, and Artery Calcification in LDLR-/- Mice Fed with High Fat Diet via Down-regulation of Tumor Necrosis Factor-α and TNF Receptor 1
Source: PLoS One. 2015 Dec 1;10(12):e0143686. doi: 10.1371/journal.pone.0143686 (PMC4666466; doi:10.1371/journal.pone.0143686)

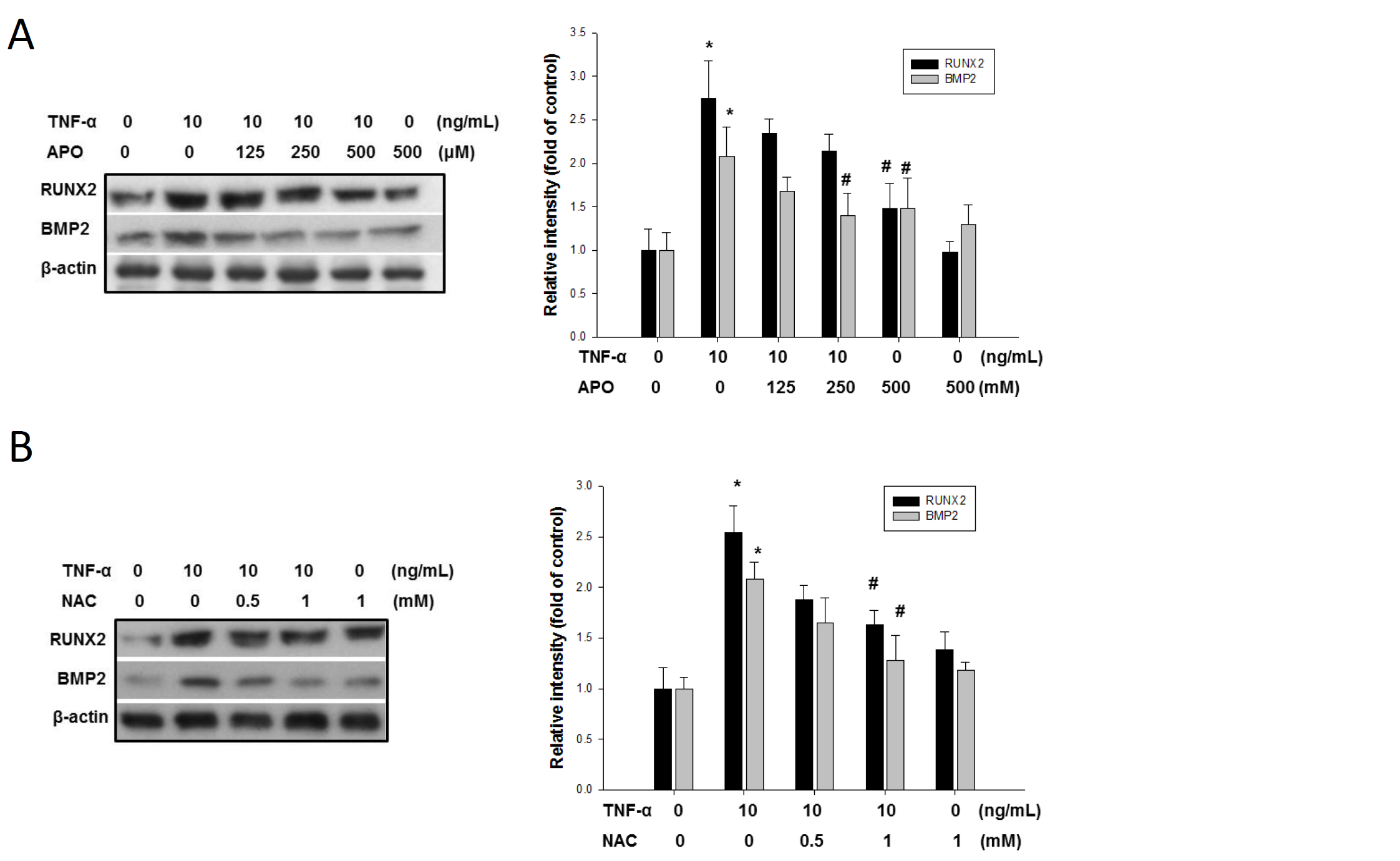

Supplement: S1 Fig — HASMCs were cultured in osteogenic differentiation medium for 3 day in the presence or absence of TNF-α (10 ng/mL) concomitantly with apocynin (A) or NAC (B). *p<0.05 compared to the control group, and #p<0.05 compared to the TNF-α groups. N = 6 for each set of experiments. (TIF) [file pone.0143686.s001.tif]

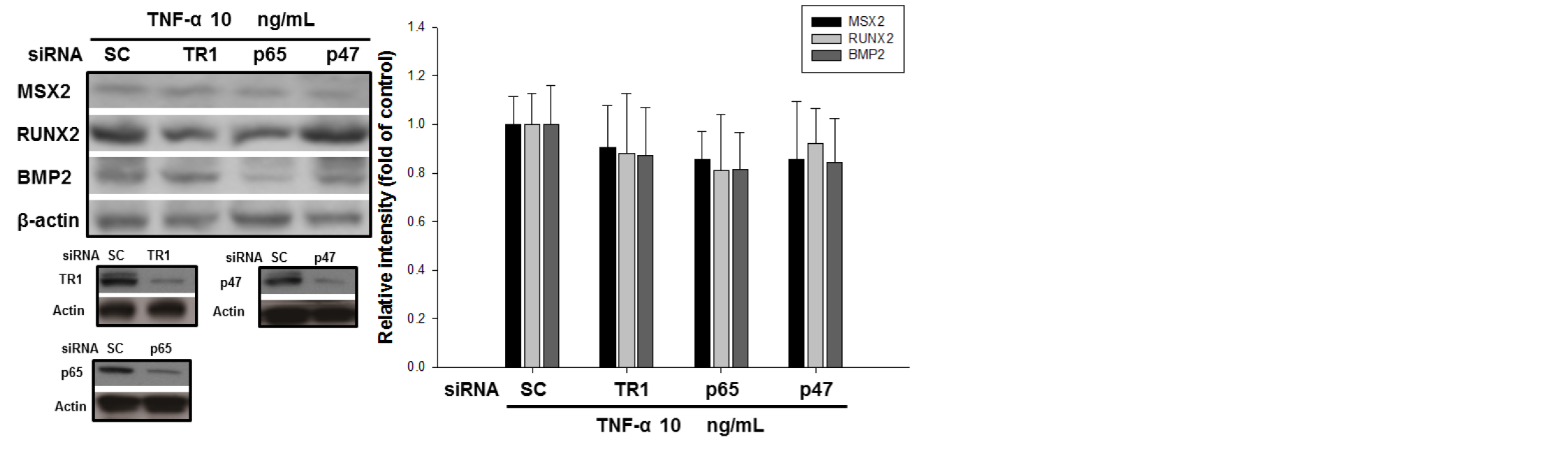

Supplement: S2 Fig — Western blotting assay the effects of the knock-down of the TNFR1, p65 and p47 proteins by these siRNAs indicated that they were functioning. Compared with the TNF-α-stimulated cells in the presence of scrambled siRNAs, any of the TNFR1, p65 or p47 siRNAs, didn’t dramatically abolished TNF-α-stimulated bone marker expression in the HASMCs. N = 6 for each set of experiments. (TIF) [file pone.0143686.s002.tif]
